# Enhanced production of prodigiosin by *Serratia marcescens* MO-1 using ram horn peptone

**DOI:** 10.1590/S1517-838246246220131143

**Published:** 2015-06-01

**Authors:** Esabi Basaran Kurbanoglu, Murat Ozdal, Ozlem Gur Ozdal, Omer Faruk Algur

**Affiliations:** 1 Atatürk University, Department of Biology, Faculty of Science, Ataturk University, Erzurum, Turkey, Department of Biology, Faculty of Science, Ataturk University, Erzurum, Turkey.; 2 Atatürk University, Department of Food, Ispir Hamza Polat Vocational School, Ataturk University, Erzurum, Turkey, Department of Food, Ispir Hamza Polat Vocational School, Ataturk University, Erzurum, Turkey.

**Keywords:** prodigiosin, *Serratia marcescens*, ram horn peptone, agricultural waste

## Abstract

This work addresses the production of prodigiosin from ram horn peptone (RHP) using MO-1, a local isolate in submerged culture. First, a novel gram-negative and rod-shaped bacterial strain, MO-1, was isolated from the body of the grasshopper (*Poecilemon tauricola* Ramme 1951), which was collected from pesticide-contaminated fields. Sequence analysis of 16S rDNA classified the microbe as *Serratia marcescens*. The substrate utilization potential (BIOLOG) and fatty acid methyl ester profile (FAME) of *S. marcescens* were also determined. The effect of RHP on the production of prodigiosin by *S. marcescens* MO-1 was investigated, and the results showed that RHP supplementation promoted the growth of MO-1 and increased the production of prodigiosin. A concentration of 0.4% (w/v) RHP resulted in the greatest yield of prodigiosin (277.74 mg/L) after 48 h when mannitol was used as the sole source of carbon. The pigment yield was also influenced by the types of carbon sources and peptones. As a result, RHP was demonstrated to be a suitable substrate for prodigiosin production. These results revealed that prodigiosin could be produced efficiently by *S. marcescens* using RHP.

## Introduction

Natural pigments are synthesized by living organisms, such as plants, animals, fungi and bacteria. Prodigiosin (C_20_H_25_N_3_O) is a linear tripyrrole red pigment and bioactive secondary metabolite that accumulates on cell membranes and intracellular granules. Although *S. marcescens* is the major producer of prodigiosin, this pigment is also produced by other bacteria, such as *Streptomyces coelicolor*, *S. lividans*, *Hahella chejuensi*, *Pseudovibrio denitriccans*, *Pseudoalteromonas rubra*, P. denitrificans, *Vibrio gazogenes*, *V. psychroerythreus*, *Serratia plymuthica* and *Zooshikella rubidus* (D'Aoust and Gerber, 1974; Kim *et al.*, 2007; Lee *et al.*, 2011; El-Bondkly *et al.*, 2012).

Prodigiosins have recently received renewed attention for their antialgal, antibacterial, antifungal, antimalarial, antiprotozoal, anticancer, immunosuppressive, antiproliferative and UV-protective activities (Montaner and Perez-Thomas, 2001; Soto-Cerrato *et al.*, 2004; Boric *et al.*, 2011; Lee *et al.*, 2011; Park *et al.*, 2012); however, its insecticidal activity was also reported (Wang *et al.*, 2011). Because of its potential use in medical and textile applications (Siva *et al.*, 2012), prodigiosin is attracting an increasing interest.

The production of prodigiosin has been shown to be influenced by many factors, such as species type and environmental factors, including inorganic phosphate availability, dissolved oxygen level, light, media composition, temperature, pH and incubation time (Williams *et al.*, 1971; Witney *et al.*, 1977; Solé *et al.*, 1997; Slater *et al.*, 2003; Wang *et al.*, 2011; Ryazantseva *et al*,. 2012). A cost reduction in prodigiosin production could be achieved using inexpensive substrates. Potential substrates, such as vegetable oils (Giri *et al.*, 2004; Wei and Chen, 2005), ethanol (Cang *et al.*, 2000), cassava wastewater, corn steep liquor (Araújo *et al.*, 2010) and squid pen powder (Siva *et al.*, 2012), have been investigated.

Many studies demonstrated that inorganic nitrogen sources, especially ammonium salts, such as (NH_4_)_2_SO_4_, NH_4_Cl, NH_4_NO_3_ and also urea, inhibit the production of the pigment, because ammonium is a poor nitrogen atom donor and perhaps indicating the toxicity of ammonium salts towards the organism (Hejazi and Falkiner, 1997; Cang *et al.*, 2000). Therefore, there exists a need for new, available and cheap organic nitrogen sources. Moreover, organic nitrogen sources such as peptones contain different amino acids, which increase the production of prodigiosin. Ram horns, which largely consist of fibrous proteins, are significant waste products of the meat industry and are produced in many areas of the world. For example, the slaughterhouses in Turkey directly discharge approximately 600 tons of ram horns per year (Kurbanoglu, 2004a). In previous studies, the addition of ram horn hydrolysate to a substrate enhanced the production of citric acid (Kurbanoglu and Kurbanoglu, 2004), xanthan gum (Kurbanoglu and Kurbanoglu, 2007) and lactic acid (Kurbanoglu, 2004b). In the current study, RHP was added as a supplement during the production of prodigiosin. The aim of the present study was to utilize RHP (as a complete substrate and nitrogen source) in submerged culture for the production of prodigiosin by our local isolate, *S. marcescens* MO-1, to enhance prodigiosin production and reutilize this abundant animal waste.

## Materials and Methods

### Materials

The chemicals, culture media and media components used in this study were purchased from Oxoid (Basingstoke, UK), Sigma-Aldrich (St Louis, MO, USA), Merck (Darmstadt, Germany) and Difco (Detroit, MI, USA). All of the reagents used were of analytical grade. RHP was reproduced according to the method of Kurbanoglu and Kurbanoglu (2004).

### Isolation and identification of *S. marcescens* strain MO-1

*S. marcescens* MO-1 capable of producing chitinase was isolated by Okay *et al.* (2013) from fields contaminated with pesticides. Preliminary identification of MO-1 was performed using MIS (Microbial Identification System) to analyze the fatty acids and BIS (Biolog Identification System) to analyze the substrate utilization capacity. The analysis of the fatty acids of MO-1 was performed according to the method described by the manufacturer's manual (Sherlock Microbial Identification System version 4.0, MIDI, Inc., Newark, DE, USA). FAMEs were separated by GC with a fused-silica capillary column (25 m × 0.2 mm) with cross-linked 5% phenyl methyl silicone. The FAME profile of the bacterial strain was identified by comparing the commercial databases with the MIS software package. The BIOLOG tests were performed using substrate plates designed from gram-negative bacteria. MO-1 was verified by the analysis of the16S rDNA sequence (Refgen Life Sciences, Ankara, Turkey) which was compared with the National Center for Biotechnology Information (NCBI) database using the web-based BLAST program (http://www.ncbi.nlm.nih.gov/BLAST) and resubmitted to GenBank (Accession Number: JX315621.2). The isolate was named as *S. marcescens* strain MO-1.

### Media and culture conditions

One loop of cells grown on NA plates for two days was used to inoculate a 250 mL flask containing 50 mL of Nutrient Broth (NB; Merck). For prodigiosin production, the bacteria were grown in NB at 28 °C and 200 rpm for 18 h. One milliliter (2%) of culture was added to 50 mL of control and production media in a 250 mL flask and incubated in a shaker at 200 rpm and 28 °C. The control medium (CM) was composed of yeast extract 0.4% (w/v) and 1% (w/v) D-Mannitol. To determine the effects of RHP on prodigiosin production, 0.1–0.6% (w/v) RHP were added to the CM. The pH was adjusted to 7 before autoclaving at 121 °C for 20 min. Later, RHP was compared with three commercial peptones (tryptone (TP), bacto peptone (BP), fish peptone (FP) at the optimal RHP concentration. For the experiments exploring the effect of carbon sources on prodigiosin production, the mannitol in the CM was replaced by glycerol (1%, v/v) and glucose (1%, w/v).

### Analytical methods

The prodigiosin content was measured using UV-spectrophotometry and was expressed in mg/L. For this purpose, the supernatant of the culture broth (1 mL) was centrifuged at 10,000 rpm for 10 min. The supernatant was discarded and the pellet was resuspended in acidified methanol (4.0 mL of 1 N HCI - 96.0 mL of methanol) to extract prodigiosin from the cells. The cell debris was removed by a second centrifugation step and the supernatant was transferred to a spectrophotometer cuvette for absorbance measurements. The prodigiosin content of the supernatant was measured by spectrophotometry at 535 nm and compared to a standard curve prepared with a prodigiosin extract (Giri *et al.*, 2004). The biomass was determined following centrifugation at 5,000 rpm for 20 min at 4 °C, drying the cell mass at 80 °C overnight and weighing the resulting dry cell biomass.

### Statistical analysis

The statistical analyses of the data were performed using one-way analysis of variance (ANOVA). The level of significance was p < 0.05. All of the statistical analyses were performed using the SPSS 15.0 software program. All of the experiments were performed in triplicate and the standard deviation (±) was calculated.

## Results and Discussion

### Bacterial strain and its characterization

**Figure 1:**
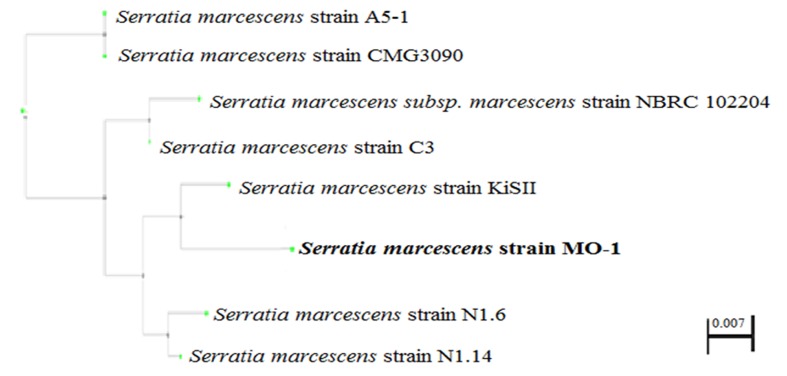
Phylogenetic tree based on the 16s rDNA sequences of strain MO-1 and related species.

MO-1 formed red-pigmented smooth colonies on nutrient agar plates. Classical tests showed that it was a gram-negative, rod-shaped and mobile organism. The results of the MIS analysis identified the MO-1 as *Serratia marcescens* with a percentage of 96.25%. The cellular fatty acids of MO-1 and some other isolates (ZJ-C0701 and ZJ-S0801) are given in Table 1. As shown in Table 1, 18 different fatty acids were detected in the MO-1 strain. Fifteen of them (10:0; 10:0 3OH; 12:0; 12:0 20OH; 12:0 3OH; 14:0; 14:0 2OH; 14:0 3OH; 15:0; 16:0; 16:0 3OH; 17:0; 17:0 cyclo; 18:0; and 19:0 cyclo) are saturated fatty acids. Palmitic acid (16:0; n-hexadecanoic acid) had a higher relative mass compared to the remaining FAMEs, whereas 10:0; 12:0 2OH; 15:0; 16:0 3OH; 17:0; and 18:0 were found to have quite low relative mass ratios. Except for the fatty acids 12:1 30H; 15:0; and 19:0 cyclo, all of the other fifteen fatty acids were present in the cells of the three isolates; however, 12:1 3OH; 15:0; and 19:0 cyclo fatty acids were only present in the strain MO-1. The BIOLOG Microplate assay showed that MO-1 is capable of using 79 of the 95 substrates in the panel and confirmed that MO-1 displayed similarity to BIOLOG's standard identification. Partial reactions of the BIOLOG kit are presented in Table 2. Strain 90-166 and H02-A (Rascoe *et al.*, 2003) had similar utilization reactions with MO-1 for some substrates on the Biolog plates. Finally, 1450 bp 16S ribosomal DNA sequence of the strain was BLAST searched (http://www.ncbi.nlm.nih.gov/BLAST) and aligned with *S. marcescens* sequences. The sequence was deposited in GenBank with the accession number of JX315621.2. A phylogenetic tree was constructed based on the 16S rDNA sequences (Figure 1). The isolate was named as *S. marcescens* strain MO-1.

**Table 1:** The percentage of fatty acids in the isolate of MO-1 and two other strains of *S. marcescens.*

Fatty acids	Strains		
	
	MO-1	ZJ-0701^a^	ZJ-S0801^a^
10:0	0.31	0.23	0.25
10:0 3OH	14.08	3.01	2.89
12:0	1.61	1.12	1.38
12:0 2OH	0.68	0.40	0.51
12:0 3OH	4.29	1.51	1.35
12:1 3OH	3.51	0	0
14:0	4.50	4.86	5.12
14:0 2OH	1.84	3.80	2.10
14:0 3OH	6.40	7.05	8.12
15:0	0.34	0	0
16:0	25.62	30.80	28.55
16:0 3OH	0.20	0.18	0.22
16:1 w7c	11.78	11.46	12.20
17:0	0.18	1.19	1.00
17:0 cyclo	9.89	15.26	14.71
18:0	0.26	0.26	1.20
18:1 w7c	12.40	15.12	16.58
19:0 cyclo	1.47	0	0

a Li *et al.*, 2011.

**Table 2:** Partial substrate utilization results of MO-1 and two other strains of *S. marcescens* using the BIOLOG Microplate.

Substrats	MO-1	90-166^b^	H02-A^b^
α-D-Lactose	+	−	+
α-Hydroxybutric acid	+	±	+
α-Ketoglutaric acid	+	+	+
β-hydroxybuturic acid	+	+	+
Adonitol	+	+	+
Alaninamide	+	+	+
Bromosuccunic acid	+	+	+
Cellobiose	±	±	+
D, L-Lactic acid	+	+	+
D-Alanine	+	+	+
D-Arabitol	−	+	+
D-Mellobiose	+	+	+
D-Serine	+	+	+
D-Sorbitol	+	±	+
Gentibiose	+	+	+
Glucuronamide	+	+	+
Hydroxy-L-Proline	+	+	+
i-Erythritol	+	+	+
L-Alanine	+	+	+
L-Alanyl glycine	+	+	+
L-Arabinose	−	+	+
L-Aspartic acid	+	+	+
L-Fucose	+	+	+
L-Ornithine	−	±	+
L-Phenylalanine	+	±	±
m-Inositol	+	+	+
N-Acetyl-D-Galactosamine	+	+	+
Putrescine	+	+	+
Succinamic acid	+	+	±
Thymidine	+	+	+

+: positive result, -: negative results.

b Rascoe *et al.*, 2003.

### Effect of RHP on the growth of MO-1 and its prodigiosin producing potential

**Figure 2:**
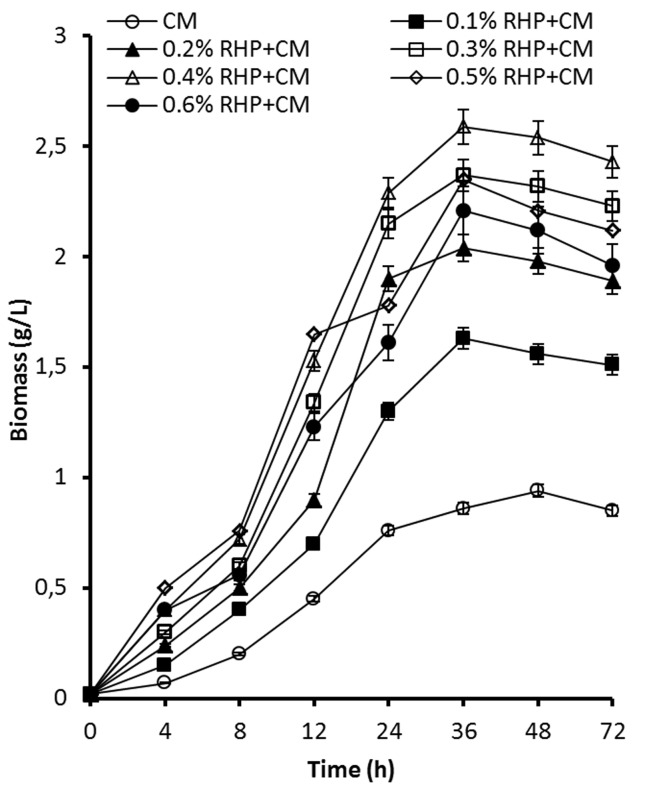
Comparison of the effect of RHP at various concentrations on biomass using mannitol as a carbon source. Culture conditions: initial pH 7.0, 200 rpm, 28 °C.

The effects of RHP on the growth of MO-1 is summarized in Figure 2. The results showed that the growth of MO-1 was very low in the control medium (CM). For example, the maximum growth yield of MO-1 in the CM was 0.94 g/L for a 48 h growth period, whereas the addition of RHP to the substrate increased the biomass yield. When the effects of different RHP concentrations on growth were compared, the maximum growth promoting effect was observed with the application of 0.4% (w/v) RHP (2.54 g/L for a 48 h growth period); however, the growth yield decreased at RHP concentrations greater than 0.5% and the lowest biomass yield (1.56 g/L) was obtained from the application of 0.1% (w/v) RHP (except for CM application).

**Figure 3:**
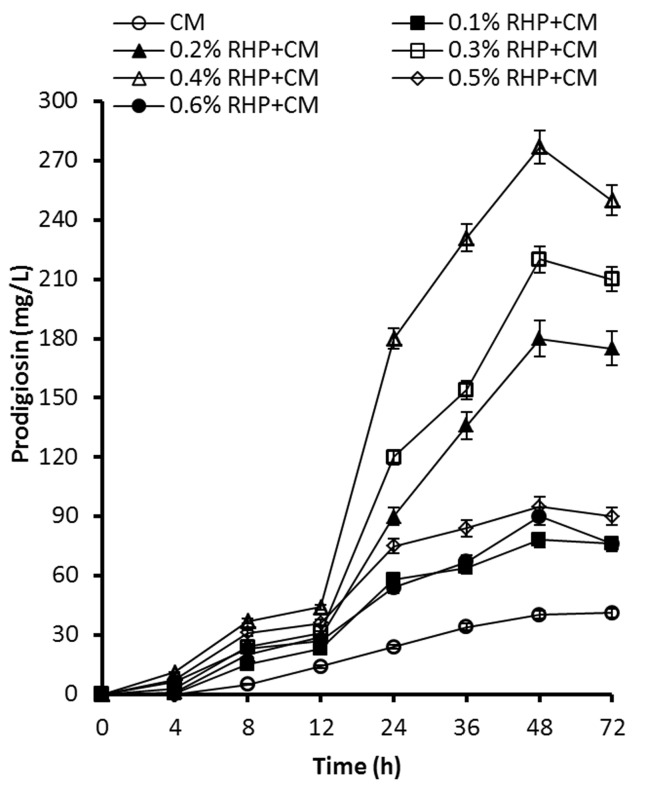
Comparison of the effect of RHP at various concentrations on prodigiosin production. Culture conditions: initial pH 7.0, 200 rpm, 28 °C.

The effects of RHP on the production of prodigiosin are presented in Figure 3. Prodigiosin is a known secondary metabolite that does not have a role in growth, development and reproduction, as do the primary metabolites, and is typically formed during the end or near the stationary phase of growth. Observed together, Figures 2 and 3 reveal that the production of prodigiosin increases linearly between 12 and 36 h. Additionally, the characteristics of the production curve are similar to the production pattern of secondary metabolites. According to Figures 2 and 3, biomass and prodigiosin production increase with increasing RHP concentrations up to 0.4%; beyond 0.4% RHP concentrations (at 0.5 and 0.6% RHP) the productions decrease.

Similar to the growth of MO-1, the production of prodigiosin was significantly (p < 0.05) affected by the addition of RHP and the maximum prodigiosin yield (277.74 mg/L) was obtained with the application of 0.4% (w/v) RHP.

The yield produced with the application of 0.4% RHP was more than 7 times greater than that obtained in the CM (40.12 mg/L). This prodigiosin production promoting effect of RHP may be due to many factors related to the chemical composition of RHP. We previously analyzed (Kurbanoglu and Kurbanoglu, 2004) the chemical composition of RHP and determined that it is very rich in both organic and inorganic nutrients and contains many amino acids and inorganic materials (data are not given). Others have reported that some amino acids (alanine, serine, histidine, cystine, methionine, glutamate, tryptophan and proline) contribute to the formation of prodigiosin (Williams *et al.*, 1971; Qadri and Williams, 1973; Witney *et al.*, 1977). The results of our previous studies (Kurbanoglu and Kurbanoglu, 2004; Kurbanoglu 2004b; Kurbanoglu and Kurbanoglu, 2007) showed that RHP contains all of these amino acids at varying concentrations and is especially rich in alanine, cysteine, glutamate and proline.

Trace elements are known to have important effects on secondary metabolites. RHP also contains some of the trace elements such as Fe, Mn, Zn, Mg and Cu, and consequently offers a rich source for culture media. As a high concentration of phosphate is known to have an inhibitory effect on prodigiosin yield (Witney *et al.*, 1977) and RHP is poor in inorganic phosphate, we did not use phosphate salts in the preparation of RHP. In addition, some investigators have reported that Ca, Mg, K, Na, Cl (Lewis and Corpe, 1964) and Fe (Silverman and Munoz, 1973) ions are necessary for the pigmentation produced by *S. marcescens*. As a result, the prodigiosin production promoting effect of RHP may be due to its organic and inorganic richness.

The carbon/nitrogen ratio (C/N) of a substrate plays a vital role in the production of different metabolites. Some researchers reported that prodigiosin production may also be affected by the C/N ratio (Wei and Chen, 2005; Wang *et al.*, 2011; Siva *et al.*, 2012). As mentioned above, RHP is rich in amino acids, which are the main reservoir of nitrogen in living tissues and cells. For this reason, as the RHP ratio in the media increased, the C/N decreased. In the light of this information, we can speculate that the addition of 0.4% RHP to the CM may provide the proper C/N ratio for prodigiosin biosynthesis; however, RHP addition decreased both biomass and prodigiosin yields at concentrations above 0.4%. Our previous studies also showed that, at high concentrations, RHP decreased the production yields of citric acid (Kurbanoglu and Kurbanoglu, 2004), xanthan (Kurbanoglu and Kurbanoglu, 2007), and lactic acid (Kurbanoglu, 2004b). This inhibitive effect may be due to its high salt concentration and unknown toxic materials.

### Effect of different peptones on the production of prodigiosin

The concentration and type of nitrogen sources are very important for the growth and pigment production of *S. marcescens*. Four peptones were tested for the production of prodigiosin (Table 3). The production of prodigiosin in the culture medium by *S. marcescens* MO-1 using RHP was better than the prodigiosin production using TP, BP and FP under the same conditions (p < 0.05). Kurbanoglu and Kurbanoglu (2004) reported that the nitrogen contents of RHP, BP, TP and FP are 8.0, 13.8, 10 and 10.1 (g/100 g), respectively. Chemical analysis of the peptones showed that FP, TP and BP were richer in nitrogen than RHP; however, the amino acid and rich mineral content of RHP stimulated cell growth and prodigiosin biosynthesis in *S. marcescens* MO-1. This result indicates that the nutrients in RHP are suitable for microbial growth and prodigiosin production.

**Table 3:** The effect of peptones on prodigiosin and biomass yields of *S. marcescens* MO1.

Medium	Biomass (g/L)	PG (mg/L)
CM	1.32 ± 0.08^c^	40.12 ± 2.01^e^
CM+RHP	2.54 ± 0.09^a^	277.74 ± 7.26^a^
CM+FP	2.48 ± 0.07^a^, ^b^	232.49 ± 5.6^c^
CM+ TP	2.31 ± 0.07^b^	213.14 ± 11.49^d^
CM+ BP	2.62 ± 0.14^a^	252.02 ± 8.95^b^

FP: Fish Peptone, TP: Tryptone, BP: Bacto Peptone.

Culture conditions: Initial pH 7.0, 200 rpm, 28 °C, 48 h.

Values with the same letter are not significant (p < 0.05).

### Effect of different carbon sources on the production of prodigiosin

*S. marcescens* MO-1 demonstrated an ability to utilize different carbon sources for growth and prodigiosin production but to different extents (Table 4). When glucose was supplied as a carbon source, *S. marcescens* MO-1 significantly decreased the production of prodigiosin. Some carbon sources, such as glucose and maltose, had a repressive effect on prodigiosin synthesis because they lowered the pH of the medium or repressed catabolites (Gargallo *et al.*, 1987; Sole *et al.*, 1997). Moreover, in production medium containing glucose, *S. marcescens* may produce the glucose-6-phosphate dehydrogenase alloenzyme, which inhibits pigment production (Gargallo *et al.*, 1987). *S*. *marcescens* grown on mineral media did not produce pigment when the carbon source was glucose or the nitrogen source was ammonium chloride, though proline was present in the medium (Hejazi and Falkiner, 1997). Glycerol and mannitol were used as carbon sources in a production medium, which resulted in a better yield of prodigiosin. In this study, *S*. *marcescens* produced 184.32 mg/L and 277.74 mg/L of prodigiosin in glycerol and mannitol containing medium, respectively (Table 4). Mannitol was found to be suitable for growth and prodigiosin production (Araújo *et al.*, 2010) instead of glycerol (p < 0.05). Other studies have reported different results associated with prodigiosin production. Wei and Chen (2005) observed 56-790 μg/mL of prodigiosin cultured in oil supplemented Luria-Bertani broth medium. The production of prodigiosin was reported in mutant *S. marcescens* 02 at a concentration of 96.5-583 mg/L (Tao *et al.*, 2005). Additionally, Gutierrez-Roman *et al.* (2012) reported a production of prodigiosin of 60 mg/L by *S. marcescens* CFFSUR-B2 cultivated in peanut medium.

**Table 4:** The effect of carbons on prodigiosin and biomass yields of *S. marcescens* MO1.

Carbons (%1, w/v)	Biomass (g/L)	PG (mg/L)
Mannitol	2.54 ± 0.09^a^	277.74 ± 7.6^a^
Glycerol	2.32 ± 0.05^b^	184.32 ± 5.9^b^
Glucose	2.07 ± 0.11^c^	6.35 ± 1.6^c^

Culture conditions: Initial pH 7.0, 200 rpm, 28 °C, 48 h.

Values with the same letter are not significant (p < 0.05).

## Conclusion

Organic nitrogen sources are known to be superior nitrogen sources for prodigiosin production compared to inorganic nitrogen sources. Therefore, there is a need for new, easily available and cheap organic nitrogen sources. Moreover, organic nitrogen sources, such as peptones contain different amino acids, which increase the production of prodigiosin production as described above. As is known, pollution continues to increase due to agricultural and industrial wastes. Consequently, these waste materials can be converted to highly valued commercial products, such as prodigiosin. The present study demonstrated that RHP significantly increased the production of prodigiosin from *S. marcescens* because of its high mineral and amino acid contents. Using RHP to produce prodigiosin can be cost-effective and environmentally beneficial.
